# Stability Studies of Antipseudomonal Beta Lactam Agents for Outpatient Therapy

**DOI:** 10.3390/pharmaceutics15122705

**Published:** 2023-11-30

**Authors:** Beatriz Fernández-Rubio, Laura Herrera-Hidalgo, Arístides de Alarcón, Rafael Luque-Márquez, Luis E. López-Cortés, Sònia Luque, José María Gutiérrez-Urbón, Aurora Fernández-Polo, Alicia Gutiérrez-Valencia, María V. Gil-Navarro

**Affiliations:** 1 Unidad de Gestión Clínica de Farmacia, Hospital Universitario Virgen del Rocío/Instituto de Biomedicina de Sevilla (IBiS), 41013 Seville, Spain; beatrizfernandezrub@gmail.com (B.F.-R.); mariav.gil.sspa@juntadeandalucia.es (M.V.G.-N.); 2 Unidad de Gestión Clínica de Enfermedades Infecciosas, Microbiología y Parasitologia, Hospital Universitario Virgen del Rocío/Instituto de Biomedicina de Sevilla (IBiS), 41013 Seville, Spain; aa2406ge@yahoo.es (A.d.A.); rafaeluquemarquez@gmail.com (R.L.-M.); alicia.gutierrez.valencia@gmail.com (A.G.-V.); 3 Centro de Investigación en Red de Enfermedades Infecciosas (CIBERINFEC), Instituto de Salud Carlos III, 28029 Madrid, Spain; luiselopezcortes@gmail.com (L.E.L.-C.); sluque@psmar.cat (S.L.); 4 Infectious Diseases and Microbiology Clinical Unit, University Hospital Virgen Macarena/Department of Medicine, School of Medicine, University of Sevilla/Biomedicine Institute of Sevilla (IBiS)/CSIC, 41009 Seville, Spain; 5 Pharmacy Department, Hospital del Mar, Parc de Salut Mar, 08003 Barcelona, Spain; 6 Infectious Pathology and Antimicrobials Research Group (IPAR), Institut Hospital del Mar d’Investigacions Mèdiques (IMIM), 08003 Barcelona, Spain; 7 Unidad de Gestión Clínica de Farmacia, Complexo Hospitalario Universitario de A Coruña, 15006 A Coruña, Spain; jose.gutierrez.urbon@sergas.es; 8 Unidad de Gestión Clínica de Farmacia, Hospital Universitari Vall d’Hebron, Institut de Recerca Vall d’Hebron, 08035 Barcelona, Spain; aurora.fernandez@vallhebron.cat

**Keywords:** *Pseudomonas aeruginosa*, multidrug-resistant bacteria, beta lactams, stability, outpatient parenteral antimicrobial therapy

## Abstract

Outpatient parenteral antimicrobial therapy (OPAT) is a useful treatment strategy against *Pseudomonas aeruginosa* and other multidrug-resistant bacteria. However, it is hindered by the lack of stability data for the administration of antibiotics under OPAT conditions. Our objective was to investigate the stability of nine antipseudomonal and broad-spectrum beta lactam antibiotics (aztreonam, cefepime, cefiderocol, ceftazidime, ceftazidime/avibactam, ceftolozane/tazobactam, meropenem, meropenem/vaborbactam, and piperacillin/tazobactam) to allow the spread of OPAT programs. All the antibiotics were diluted in 500 mL 0.9% sodium chloride and stored at 4, 25, 32, and 37 °C for 72 h in two different devices (infusion bags and elastomeric pumps). The solutions were considered stable if the color, clearness, and pH remained unchanged and if the percentage of intact drug was ≥90%. All the antimicrobials remained stable 72 h under refrigerated conditions and at least 30 h at 25 °C. At 32 °C, all the antibiotics except for meropenem and meropenem/vaborbactam remained stable for 24 h or more. At 37 °C, only aztreonam, piperacillin/tazobactam, cefepime, cefiderocol, and ceftolozane/tazobactam were stable for at least 24 h. The stability results were the same in the two devices tested. All the antibiotics studied are actual alternatives for the treatment of antipseudomonal or multidrug-resistant infections in OPAT programs, although the temperature of the devices is crucial to ensure antibiotic stability.

## 1. Introduction

*Pseudomonas aeruginosa* is a gram-negative bacteria recognized for its ubiquity and its highly developed resistance mechanisms [1]. This microorganism is commonly associated with nosocomial and fatal infections in immunocompromised individuals, particularly in patients with cystic fibrosis, for whom *P. aeruginosa* is the major pulmonary pathogen [2,3]. Given its growing relevance, the World Health Organization listed *P. aeruginosa* as a priority pathogen for the research and development of new antibiotics in 2017 [4]. Treatment for *P. aeruginosa* is currently limited due to its high level of natural resistance to antibiotics and its great capacity to acquire different mechanisms of resistance via chromosomal mutations or horizontal transmission of genetic materials, with the resulting risk of challenging antibiotic therapy [5,6,7,8]. Oral antipseudomonal agents such as fluoroquinolones can be successfully used in mild infections, but severe ones frequently require the administration of intravenous antibiotics [9]. Among the available options, beta lactams have largely been the cornerstone of antimicrobial therapy against *P. aeruginosa*, as well as for many other multidrug-resistant gram-negative bacteria [10].

The inclusion of patients who suffer from an infection caused by *P. aeruginosa* in outpatient parenteral antimicrobial therapy (OPAT) programs has increased over the last years [11,12]. OPAT is usually defined as the outpatient or community-based management of an infection via the administration of an intravenous antimicrobial without an overnight hospital stay [13]. This healthcare tool provides multiple advantages, including significant hospital cost savings or readmission avoidance, as well as reducing the hospital dissemination of multidrug-resistant bacteria [14]. Additionally, it improves the patient’s quality of life, especially for those who suffer from repeated exacerbations because they can be treated in the comfortable environment of their home, avoiding multiple hospital admissions [15,16]. In OPAT programs, antimicrobials can be administered by gravity infusion or with portable pumps. In the last scenario, electronic or elastomeric devices are the two available alternatives, and both allow the use of extended infusions [17]. However, a lack of drug stability may lead to significant difficulties since patients could receive a lower dose of antibiotic than required to treat the infection. In that case, it would be necessary to prolong the duration of treatment or even switch to another antibiotic, increasing the risk of hospital readmission and treatment failure. Furthermore, antimicrobial resistance could rise since inappropriate doses are associated with the increment of multidrug-resistant bacteria [18]. Many factors have an impact on drug stability, the temperature of storage, the diluent used, the infusion container material, and the drug concentration being the most relevant [19]. Given the great disparity in the chemical structure of beta lactams and the different stability information provided, robust stability data of these antimicrobials at the conditions reached in OPAT are needed in order to increase the number of patients who could be treated in these programs [20].

The aim of this study was to assess the physicochemical stability of nine beta lactams antibiotics for the treatment of *P. aeruginosa* and other multidrug-resistant bacteria contained in infusion bags and elastomeric devices at four different temperatures in order to provide novel data for their use in the OPAT setting: aztreonam (AZT), cefepime (CEF), cefiderocol (CFD), ceftazidime (CAZ), ceftazidime/avibactam (C/A), ceftolozane/tazobactam (C/T), meropenem (MRP), meropenem/vaborbactam (MEV), and piperacillin/tazobactam (P/T).

## 2. Materials and Methods

### 2.1. Materials

AZT, CEF, CAZ, and vaborbactam (VAB) standards were obtained from MedChemExpress (Monmouth Junction, NJ, USA). Avibactam (AVI), CFD, ceftolozane (CFT), MRP, piperacillin (PIP), and tazobactam (TZB) standards were purchased from Alsachim (Illkirch, France), as well as the internal standards, ampicillin (AMP) and cefixime (CFM).

Pharmaceutical dosages were prepared using the following commercial intravenous formulations:

- Aztreonam 1 g (Azactam^®^) (Bristol-Myers Squibb, Tokyo, Japan).
- Cefepime Torlan 1 g and 2 g (LDP Laboratorios, Barcelona, Spain).
- Cefiderocol 1 g (Fetcroja^®^) (Shionogi, Osaka, Japan).
- Ceftazidime Qilu 1 g (Qilu Pharma Spain, Madrid, Spain) and Ceftazidime Sala 2 g (Lab. Reig Jofre, S.A., Barcelona, Spain).
- Ceftazidime/Avibactam 2 g/0.5 g (Zavicefta^®^) (Pfizer, Williamsburgh, NY, USA).
- Ceftolozane/Tazobactam (Zerbaxa^®^) 1 g/0.5 g (MSD, Rahway, NJ, USA).
- Meropenem SUN 1 g (Sun Pharmaceutical Industries, Mumbai, India).
- Meropenem/Vaborbactam (Vaborem^®^) 1 g/1 g (Menarini International O.L. S.A., Florence, Italy).
- Piperacillin/tazobactam 4 g/0.5 g (Aurovitas, Madrid, Spain) and Piperacillin/tazobactam Kabi 2 g/0.25 g (Fresenius Kabi, Bad Homburg, Germany).

Sterile water for injection used for the reconstitution of the drug vials was purchased from Serra Pamies Laboratories (Tarragona, Spain). For the preparation of the solution tests, drugs were stored in polypropylene infusion bags obtained from Chirana T. Injecta (Trenčín, Slovakia) and in polyisoprene elastomeric devices that were supplied by Leventon (Barcelona, Spain).

Liquid chromatography-mass spectrometry (LC-MS) grade (reagent grade, >98% pure) acetonitrile was obtained from Merck KGaA (Darmstadt, Germany), and formic acid was purchased from Scharlab (Barcelona, Spain). Ammonium formate was obtained from Acros Organics (Morris Plains, NJ, USA). Purified water was obtained from a Milli-Q Academic ultrapure water system (Millipore Corp., Bedford, MA, USA).

### 2.2. Choice of Concentrations

A team made up of infectious disease specialists and antimicrobial hospital pharmacists with clinical experience in OPAT determined the total daily dose and the maximum volume to be administered at home in 24 h, 500 mL. These considerations were used to obtain the final concentrations of each antibiotic, which are summarized in Table 1.

### 2.3. Preparation of Solutions

Each antibiotic was reconstituted with water for injection to obtain a concentration of 100 g/L. These solutions were further diluted in 0.9% sodium chloride to obtain the final concentration displayed in Table 1 and subsequently introduced individually into the containers. Three bags and three elastomeric pumps for each temperature condition and for each antibiotic were prepared.

### 2.4. Storage Conditions and Sampling

Bags and elastomeric devices were stored protected from light at 4 different temperatures: refrigerated (4 ± 2 °C), 25 ± 2 °C, 32 ± 2 °C, and 37 ± 2 °C. Different analysis samples were taken over 72 h (0, 12, 24, 30, 48, and 72 h). At each timepoint, duplicate samples from every preparation were collected and frozen at −80 °C until the analysis. Before the chemical analysis, samples were diluted in Milli-Q water, vortexed, aliquoted in autosampler vials, and injected into the HPLC-MS/MS.

### 2.5. LC-MS/MS Quantification

Antibiotic concentrations were measured by a liquid chromatography-tandem mass spectrometry (LC-MS/MS) method developed for each drug. Samples were analyzed using an Agilent 1290 Infinity liquid chromatograph (Agilent Technologies, Palo Alto, CA, USA) coupled with an AB SCIEX API 4000 mass spectrometer operating in electrospray positive or negative ionization mode, depending on the drug. Nitrogen was used as the collision gas. AMP was used as the internal standard for AVI and AZT, while CFM was the internal standard for the quantification of CEF, CAZ, PIP, TZB, CFT, and MRP. Chromatographic and mass spectrometry conditions of each method are detailed in the supplementary material. Validation of the method was performed following the U.S. Food and Drug Administration guidelines [21].

### 2.6. Chemical Stability

Drug stability was calculated as the percentage (*P*) of the initial drug concentration remaining in the device at each analyzed time point (*Ct*) in relation to the concentration at the initial time (*C*0) (*P* = *Ct*/*C*0 × 100). Chemical stability was defined as the recovery of more than 90% of the initial concentration of the antibiotic [21]. Data are expressed as mean and 90% confidence interval (CI).

### 2.7. Physical Stability

Color change, clearness, and precipitation were assessed by visual inspection with the unaided eye at each sampling time point. pH was determined at each analysis time point using a stainless electrode pH meter (Hach, Düsseldorf, Germany). Physical changes observed in the experiments and described in the summary of product characteristics (SmPC) of each drug, such as color changes, were considered acceptable. A variation of more than one pH unit was considered physically unstable [22].

## 3. Results

### 3.1. Chemical Stability

At the refrigerated temperature (4 °C), all the antibiotics remained chemically stable for the whole experiment (72 h). At 25 °C, CEF, CAZ, and C/A were stable for 48 h, while MRP and MEV maintained stability during 30 h of storage. The rest of the antibiotics (AZT, CFD, C/T, and P/T) remained stable for 72 h. At 32 °C, CEF was stable for 48 h, CAZ and C/A for 30 h, CFD for 24 h, and MRP and MEV for 12 h. The remaining antimicrobials (AZT, C/T, and P/T) attained the stability criterion of ≥90% of the original concentration for the entire experiment. Regarding the highest temperature, 37 °C, AZT and P/T were stable for 72 h, while the rest of the antibiotics remained stable until different analyzed time points: CEF and CFD for 24 h, CAZ and C/A for 12 h, C/T for 48 h, and MRP and MEV were not stable at any time point. The chemical stability results described were the same in the two administration devices at the four temperatures tested.

The percentages and 90% CIs of the remaining concentrations that were obtained at each analytic time point during 72 h at 4 °C, 25 °C, 32 °C, and 37 °C in both devices are listed in Table 2, Table 3, Table 4, and Table 5, respectively.

### 3.2. Physical Stability

There were no observed changes in the color of any solution of AZT, CFD, CAZ, C/A, and MRP. At 25 °C, 32 °C, and 37 °C, CEF, C/T, MRP, and MEV ranged from colorless to a faint yellowish, dark yellow, or even slightly orange in some conditions. The color changes observed were detailed in the SmPc. No visible precipitation was observed for all samples from both devices at any temperature, and all samples appeared clear with no visible turbidity. The baseline pH was generally stable with a change of less than one unit except for CEF at 32 °C after 48 h and 37 °C after 24 h and CFD at 37 °C after 24 h in both devices. Therefore, CEF and CFD were physically unstable from that time point at the temperatures described. Table 6 summarizes the physical stability obtained during 72 h at 4 °C, 25 °C, 32 °C, and 37 °C in both infusion bags and elastomeric pumps.

Table 7 shows the global stability of each beta lactam at the four temperatures studied.

## 4. Discussion

The present study provides useful information regarding the stability of nine parenteral beta lactams, including penicillins, cephalosporins, carbapenems, and monobactams, with potential use in severe infections caused by *P. aeruginosa* and other gram-negative organisms through continuous infusion. These data are essential in order to encourage the utilization of OPAT programs, which avoid the high risk of nosocomial infections produced by multidrug-resistant organisms [23].

Over the last few years, the prolonged beta lactam infusion strategy has been established as the standard method for the administration of this group of antibiotics [24,25]. Given that they are time-dependent drugs, their killing activity is related to the maintenance of the free concentration exceeding the minimum inhibitory concentration (%free T > MIC), so prolonged infusions may attain the pharmacodynamic target more effectively than intermittent infusions. In consequence, the administration of beta lactam antibiotics by extended or continuous infusions, rather than standard administrations over approximately 30 min, has been associated with improved clinical outcomes [26]. This is particularly relevant in multidrug-resistant microorganisms such as *P. aeruginosa* since a continuous infusion strategy could reduce the probability of breakthrough infections and achieve successful outcomes. Therefore, using high-dose continuous infusion of beta lactam antibiotics to target high drug concentrations at or above the MIC of resistant *P. aeruginosa* infections has become a potentially useful treatment for optimal bacterial killing and microbiologic response [27,28]. Our results show that these drugs can be administered at home via continuous infusion (except for CAZ and C/A at 37 °C and MRP and MEV at 32 °C and 37 °C), which allows for maximizing the efficacy of the treatment against potentially multidrug-resistant pathogens in an environment in which their transmissibility is minimized.

In consequence, drug stability is crucial for the administration of beta lactams using continuous infusion in OPAT programs. It depends upon four essential factors: concentration, diluent, infusion delivery device, and storage temperature [29]. In the present investigation, the choice of the concentration of the studied beta-lactams was decided based on the maximum daily dose approved for each antibiotic, that is, the usually recommended dose for the treatment of multidrug-resistant microorganisms using continuous infusion and the highest volume that is typically administered ambulatory in 24 h using the most usual diluent, 0.9% sodium chloride, in order to avoid complications in the vascular access, such as phlebitis. Regarding the infusion device, this stability study was carried out using electronic infusion pumps and elastomeric devices, which are the most commonly used in OPAT programs due to several advantages. Electronic infusion bags use a positive pumping action, so they provide an accurate flow of drugs over a prescribed period, and they are usually equipped with safety features, such as alarms. On the contrary, elastomeric devices are light, silent, and do not require an external power supply for their functioning, allowing for the complete mobility of the patient [30,31]. Our investigation has found the same stability results in both devices when other parameters such as external temperature and concentration are identical, although it is known that the composition of the devices could have an impact on drug stability [32]. The last fundamental factor over drug stability is the external temperature, especially within the 20 °C to 37 °C range, since it is well-known that an increment in temperature leads to an increase in drug degradation [33]. When administered at home, the temperature is not usually under control, and high temperatures are commonly achieved, so it is an essential parameter that must be taken into account. Elastomeric devices are particularly affected by external temperature because they are placed next to the body, so temperatures as high as 32 °C or even 37 °C may be easily achieved [18,34]. However, most stability data come from studies performed at room temperature (25 °C) but not at higher temperatures. Therefore, to evaluate preparation and storage feasibility, we appraised the stability of the antibiotics at 4 °C, and room (25 °C), 32 °C, and 37 °C temperature conditions were also assessed in order to simulate the home environment.

Eradication of *P. aeruginosa* has become steadily more difficult due to its remarkable capacity to resist antibiotics, so we have studied the stability of almost all the antipseudomonal antibiotics commercialized in our country in order to obtain a wide variety of therapeutic alternatives in OPAT. There are a few exceptions like imipenem, whose instability is well-defined, ciprofloxacin and levofloxacin, which can be administered in the oral form, and antimicrobials that usually cause significant nephrotoxic effects that need therapeutic drug monitoring (which is difficult in the home environment), such as aminoglycosides or colistin [35,36,37]. However, this study provides useful information for the OPAT setting, including the novel antipseudomonal agents CAV, C/T, and even CFD and MEV, recently approved antibiotics with limited published studies on their stability. Additionally, it includes aztreonam, which is generally recommended to beta lactams-allergic patients [38]. Since our results have demonstrated that the nine beta lactam antibiotics tested are stable for 72 h under refrigerated conditions, sequential refrigerated storage for 24 or 48 h followed by the 24 h period of administration may be a potential strategy to provide patients with up to three days’ worth of antibiotics in a single delivery. In consequence, OPAT programs would reduce costs associated with nursing visits and pharmacy drug preparation, and they may allow at least twice as many patients to be treated without an increment of the resources needed. Nevertheless, it is imperative to be aware that MRP and MEV are not stable for more than 12 h at 32 °C, even less at 37 °C, and CAZ and C/A are also stable at a maximum of 12 h at 37 °C, so the strategy proposed cannot be applied within these antibiotics at the temperatures mentioned.

Among the strengths that can be found in our study, the technique employed to measure the concentrations of the different antibiotics was HPLC coupled with tandem mass spectrometry (MS/MS), which is inherently more sensitive and specific than other detectors, such as ultraviolet [39,40]. Not only chemical stability but also physical stability was investigated, providing valuable information about pH and color changes, which may be considered for the administration. Last but not least, the composition of the two infusion devices investigated, polypropylene in the infusion bags and polyisoprene in the elastomeric pumps, is the most commonly used at the present time, so the stability information provided can be applied in most of the OPAT programs worldwide [41]. Our investigation also has some limitations: First of all, degradation products, impurities, leachable or extractable products were not measured. This is especially relevant for the antibiotic ceftazidime, given that it is hydrolyzed to pyridine, a potential toxin. In order to administer ceftazidime through continuous infusion in OPAT minimizing pyridine formation, it has been proposed once-daily changes in the infusion device [42]. Since we could not measure the amount of pyridine produced during this study, this recommendation should be followed, although our stability results are longer than 24 h at 4, 25, and 32 °C. Secondly, the most unstable antibiotics, like MER, CFD, or CEF, could have been diluted using a citrate buffer to obtain a pH of the solution near 7 in order to enhance the stability, but this approach was not carried out [43,44]. Nevertheless, this strategy is not common in routine clinical practice given that it involves a significant manipulation of sterile solutions and therefore a considerable high risk of contamination.

To summarize, this study provides valuable data regarding the long-term stability of nine beta lactams at different temperatures with activity against *P. aeruginosa* and other multidrug-resistant bacteria. AZT and P/T were the most stable antibiotics studied, followed by C/T, CEF, and CFD, which were physically and chemically stable for at least 24 h at the four temperatures tested. CAZ and C/A remained stable for more than 24 h at 32 °C but just 12 h at 37 °C, and MRP and MEV were the least stable antimicrobials, especially at the highest temperatures tested. The container type, polypropylene infusion bags, and polyisoprene elastomeric pumps did not have an influence on the stability results, as opposed to the temperature of the devices, which was shown to be crucial to ensuring antibiotic stability. This information is crucial in order to establish and spread OPAT under real conditions and thus prevent the spread of multi-resistant strains, which have become a vitally important public health threat.

## Figures and Tables

**Table 1:** Total daily dose and concentration of every analyzed antibiotic.

Antibiotic	Total Daily Dose (g)	Concentration (g/L)
**AZT**	6	12
**CEF**	6	12
**CFD**	6	12
**CAZ**	6	12
**C/A**	6/1.5	12/3
**C/T**	6/3	12/6
**MRP**	6	12
**MEV**	6/6	12/12
**P/T**	16/2	32/4

Abbreviations: AZT, aztreonam; CEF, cefepime; CFD, cefiderocol; CAZ, ceftazidime; C/A, ceftazidime/avibactam; C/T, ceftolozane/tazobactam; MRP, meropenem; MEV, meropenem/vaborbactam; P/T, piperacillin/tazobactam.

**Table 2:** Chemical stability at 4 °C.

Antibiotic and Device			Concentration Remaining (90% CI)				
			12 h	24 h	30 h	48 h	72 h
**AZT**		Infusion bag	101.99 (3.07)	105.03 (1.63)	97.41 (3.04)	106.97 (2.06)	103.79 (3.62)
		Elastomeric pump	100.02 (3.08)	100.24 (3.56)	103.87 (2.45)	97.94 (1.35)	102.41 (3.06)
**CEF**		Infusion bag	95.16 (1.15)	97.92 (2.75)	102.80 (3.19)	98.75 (4.74)	101.18 (2.08)
		Elastomeric pump	96.11 (3.28)	101.08 (4.82)	98.21 (1.77)	105.90 (3.27)	96.53 (3.24)
**CFD**		Infusion bag	98.60 (2.96)	103.25 (5.29)	100.62 (3.39)	104.53 (5.39)	96.72 (6.39)
		Elastomeric pump	104.81 (3.12)	96.63 (2.84)	104.65 (3.08)	96.27 (1.70)	100.84 (4.64)
**CAZ**		Infusion bag	94.45 (3.28)	100.26 (4.97)	100.26 (3.58)	96.54 (3.31)	104.53 (2.86)
		Elastomeric pump	103.53 (0.67)	106.57 (3.02)	93.55 (2.39)	94.53 (1.59)	94.68 (3.65)
**C/A**	Infusion bag	Ceftazidime	97.40 (2.54)	102.40 (4.80)	103.42 (2.75)	96.15 (3.02)	104.41 (3.23)
		Avibactam	95.88 (3.66)	104.91 (1.69)	98.83 (4.87)	106.58 (4.26)	100.50 (3.43)
	Elastomeric pump	Ceftazidime	94.12 (2.47)	101.06 (5.18)	96.12 (4.45)	102.13 (3.42)	100.88 (0.29)
		Avibactam	100.10 (2.72)	99.52 (3.45)	100.75 (4.67)	95.11 (1.98)	97.35 (1.85)
**C/T**	Infusion bag	Ceftolozane	105.69 (0.39)	96.66 (5.08)	105.52 (4.79)	96.91 (5.46)	99.14 (3.68)
		Tazobactam	105.25 (4.18)	95.25 (4.78)	102.75 (3.34)	97.30 (2.59)	101.89 (4.87)
	Elastomeric pump	Ceftolozane	107.46 (2.47)	95.12 (4.79)	101.39 (5.28)	94.54 (0.86)	100.35 (2.14)
		Tazobactam	99.95 (1.99)	97.03 (5.48)	103.11 (2.13)	103.70 (1.34)	95.59 (1.13)
**MRP**		Infusion bag	105.57 (2.11)	98.68 (1.69)	96.74 (3.17)	96.15 (5.31)	94.68 (4.35)
		Elastomeric pump	98.52 (4.11)	103.83 (3.95)	98.47 (3.50)	99.49 (0.83)	102.29 (4.83)
**MEV**	Infusion bag	Meropenem	94.08 (3.05)	99.95 (4.87)	103.56 (1.77)	104.40 (4.19)	95.72 (2.40)
		Vaborbactam	101.99 (3.81)	98.29 (2.21)	103.75 (4.95)	106.78 (2.93)	101.17 (3.69)
	Elastomeric pump	Meropenem	105.78 (4.35)	105.72 (3.50)	100.62 (3.17)	93.82 (2.31)	96.50 (3.65)
		Vaborbactam	105.12 (4.72)	99.36 (4.51)	101.54 (3.23)	105.32 (4.05)	98.16 (5.24)
**P/T**	Infusion bag	Piperacillin	98.36 (5.74)	98.72 (3.86)	100.50 (5.15)	104.47 (2.74)	104.47 (5.07)
		Tazobactam	99.55 (4.95)	95.37 (1.95)	101.56 (3.92)	100.11 (3.27)	106.25 (1.65)
	Elastomeric pump	Piperacillin	97.53 (0.83)	106.81 (2.34)	104.89 (5.71)	105.60 (2.98)	94.32 (2.85)
		Tazobactam	98.94 (1.05)	100.21 (3.95)	102.67 (2.40)	106.87 (2.35)	97.49 (4.53)

Abbreviations: AZT, aztreonam; CEF, cefepime; CFD, cefiderocol; CAZ, ceftazidime; C/A, ceftazidime/avibactam; C/T, ceftolozane/tazobactam; MRP, meropenem; MEV, meropenem/vaborbactam; P/T, piperacillin/tazobactam; CI, confidence interval.

**Table 3:** Chemical stability at 25 °C.

Antibiotic and Device			Concentration Remaining (90% CI)				
			12 h	24 h	30 h	48 h	72 h
**AZT**		Infusion bag	101.71 (3.28)	100.29 (2.50)	99.57 (2.04)	97.80 (2.11)	95.54 (2.50)
		Elastomeric pump	99.21 (3.83)	102.48 (4.70)	103.71 (4.65)	96.48 (2.35)	94.34 (1.89)
**CEF**		Infusion bag	102.34 (5.39)	95.76 (4.41)	102.09 (4.62)	97.06 (5.26)	83.34 (5.56)
		Elastomeric pump	95.49 (3.30)	97.94 (4.07)	99.74 (2.83)	97.91 (3.59)	84.72 (3.93)
**CFD**		Infusion bag	104.85 (2.97)	95.04 (2.62)	98.55 (3.72)	97.73 (2.39)	94.41 (2.78)
		Elastomeric pump	100.54 (2.61)	95.78 (4.43)	105.34 (2.80)	100.29 (3.97)	97.83 (4.09)
**CAZ**		Infusion bag	97.87 (3.93)	98.83 (3.22)	96.94 (4.20)	95.00 (3.48)	85.56 (4.17)
		Elastomeric pump	94.97 (2.89)	96.91 (4.17)	107.63 (2.27)	96.41 (3.39)	83.43 (5.97)
**C/A**	Infusion bag	Ceftazidime	102.33 (2.29)	101.31 (1.21)	95.41 (1.81)	96.80 (2.57)	87.33 (2.53)
		Avibactam	95.08 (3.33)	95.24 (2.55)	98.71 (2.78)	104.88 (1.82)	96.52 (4.09)
	Elastomeric pump	Ceftazidime	100.44 (3.28)	102.74 (2.77)	100.64 (1.96)	101.39 (4.02)	84.84 (2.40)
		Avibactam	104.17 (4.91)	94.07 (2.55)	101.08 (3.37)	100.70 (1.67)	92.28 (2.08)
**C/T**	Infusion bag	Ceftolozane	104.59 (0.61)	98.62 (3.63)	101.58 (4.61)	106.92 (2.05)	98.30 (0.36)
		Tazobactam	102.05 (4.89)	95.52 (4.88)	97.19 (3.04)	103.21 (5.23)	96.46 (5.62)
	Elastomeric pump	Ceftolozane	97.76 (4.79)	98.02 (2.18)	94.25 (4.10)	98.96 (3.37)	95.77 (4.65)
		Tazobactam	102.53 (5.47)	102.59 (3.96)	95.59 (3.99)	102.32 (3.16)	102.35 (2.62)
**MRP**		Infusion bag	100.70 (4.40)	94.79 (3.39)	98.34 (4.65)	71.94 (1.44)	72.86 (3.59)
		Elastomeric pump	97.00 (4.76)	97.86 (3.18)	95.35 (0.52)	83.33 (3.58)	69.31 (3.09)
**MEV**	Infusion bag	Meropenem	106.02 (3.78)	103.92 (2.16)	93.73 (2.06)	81.93 (3.31)	77.94 (3.26)
		Vaborbactam	100.93 (3.74)	104.51 (4.54)	99.35 (4.83)	99.11 (4.88)	94.32 (4.24)
	Elastomeric pump	Meropenem	95.57 (2.55)	98.15 (2.92)	102.90 (4.54)	60.76 (3.30)	45.36 (3.74)
		Vaborbactam	98.33 (3.56)	99.48 (4.78)	96.01 (4.36)	97.73 (3.72)	93.62 (1.95)
**P/T**	Infusion bag	Piperacillin	107.14 (2.76)	99.83 (2.93)	105.19 (3.68)	106.61 (2.54)	99.55 (4.60)
		Tazobactam	101.82 (3.46)	102.33 (3.67)	105.24 (2.69)	103.06 (1.06)	96.40 (5.85)
	Elastomeric pump	Piperacillin	100.87 (4.81)	95.71 (5.11)	95.15 (3.22)	98.91 (3.13)	103.55 (3.01)
		Tazobactam	101.57 (3.49)	98.70 (4.79)	101.86 (4.74)	100.96 (1.93)	104.08 (3.28)

Abbreviations: AZT, aztreonam; CEF, cefepime; CFD, cefiderocol; CAZ, ceftazidime; C/A, ceftazidime/avibactam; C/T, ceftolozane/tazobactam; MRP, meropenem; MEV, meropenem/vaborbactam; P/T, piperacillin/tazobactam; CI, confidence interval. Unstable conditions are colored in red.

**Table 4:** Chemical stability at 32 °C.

Antibiotic and Device			Concentration Remaining (90% CI)				
			12 h	24 h	30 h	48 h	72 h
**AZT**		Infusion bag	98.16 (4.60)	97.00 (3.70)	96.43 (3.31)	96.59 (3.10)	97.07 (2.24)
		Elastomeric pump	99.61 (3.73)	96.52 (1.92)	94.93 (1.19)	99.12 (2.24)	95.33 (2.04)
**CEF**		Infusion bag	102.64 (3.85)	94.60 (3.97)	97.94 (3.00)	97.66 (4.91)	80.29 (3.20)
		Elastomeric pump	95.51 (4.34)	96.70 (4.38)	100.52 (3.67)	93.57 (2.59)	86.01 (2.70)
**CFD**		Infusion bag	104.13 (4.29)	96.15 (4.79)	56.30 (3.34)	62.43 (5.13)	56.45 (1.51)
		Elastomeric pump	96.23 (2.58)	100.76 (4.50)	70.03 (1.77)	71.64 (2.36)	74.78 (3.01)
**CAZ**		Infusion bag	98.99 (4.32)	97.12 (2.89)	99.03 (1.83)	85.22 (1.97)	76.93 (3.61)
		Elastomeric pump	107.03 (3.03)	95.41 (4.51)	94.48 (4.31)	73.37 (1.31)	73.04 (3.86)
**C/A**	Infusion bag	Ceftazidime	98.27 (1.81)	99.87 (2.59)	93.87 (3.26)	81.44 (2.25)	77.86 (3.22)
		Avibactam	97.01 (2.65)	100.64 (4.22)	98.92 (2.68)	99.49 (4.40)	95.20 (4.54)
	Elastomeric pump	Ceftazidime	100.92 (2.69)	95.24 (2.72)	94.37 (4.11)	82.52 (3.21)	67.60 (3.62)
		Avibactam	106.35 (2.56)	94.93 (1.99)	103.46 (3.10)	99.65 (4.59)	102.18 (4.69)
**C/T**	Infusion bag	Ceftolozane	106.97 (1.19)	102.78 (1.48)	103.79 (1.90)	103.97 (1.49)	101.08 (3.50)
		Tazobactam	98.20 (5.78)	104.97 (4.34)	102.51 (4.42)	95.05 (0.25)	97.24 (4.50)
	Elastomeric pump	Ceftolozane	107.17 (1.07)	105.99 (1.26)	101.20 (3.37)	98.72 (2.49)	96.20 (4.27)
		Tazobactam	101.23 (4.87)	96.25 (3.60)	93.71 (1.97)	95.03 (3.26)	100.23 (1.30)
**MRP**		Infusion bag	95.93 (3.82)	83.45 (4.18)	87.50 (2.73)	69.94 (3.62)	62.36 (4.39)
		Elastomeric pump	95.60 (3.03)	80.57 (3.24)	71.92 (1.47)	58.45 (3.99)	57.64 (4.42)
**MEV**	Infusion bag	Meropenem	99.48 (3.33)	69.46 (3.38)	67.93 (4.83)	61.37 (4.07)	33.13 (3.60)
		Vaborbactam	102.66 (2.55)	99.97 (5.34)	100.64 (4.92)	104.05 (3.75)	99.94 (3.93)
	Elastomeric pump	Meropenem	97.75 (2.09)	67.67 (3.95)	61.52 (4.33)	51.90 (3.03)	25.63 (2.76)
		Vaborbactam	102.51 (4.12)	103.56 (2.50)	94.40 (3.35)	97.64 (3.64)	94.12 (1.78)
**P/T**	Infusion bag	Piperacillin	107.00 (1.27)	108.51 (0.97)	107.02 (2.34)	102.39 (4.00)	95.83 (2.75)
		Tazobactam	100.10 (4.93)	100.18 (4.90)	96.04 (0.43)	103.13 (1.98)	98.91 (5.11)
	Elastomeric pump	Piperacillin	95.68 (3.21)	97.82 (0.94)	108.12 (2.18)	99.28 (1.85)	92.26 (1.38)
		Tazobactam	103.93 (0.43)	95.98 (5.22)	101.09 (3.25)	106.47 (3.86)	95.16 (3.20)

Abbreviations: AZT, aztreonam; CEF, cefepime; CFD, cefiderocol; CAZ, ceftazidime; C/A, ceftazidime/avibactam; C/T, ceftolozane/tazobactam; MRP, meropenem; MEV, meropenem/vaborbactam; P/T, piperacillin/tazobactam; CI, confidence interval. Unstable conditions are colored in red.

**Table 5:** Chemical stability at 37 °C.

Antibiotic and Device			Concentration Remaining (90% CI)				
			12 h	24 h	30 h	48 h	72 h
**AZT**		Infusion bag	96.86 (2.33)	95.68 (2.92)	95.19 (2.77)	98.24 (4.14)	97.09 (4.13)
		Elastomeric pump	100.83 (3.52)	99.50 (3.33)	98.10 (2.83)	102.12 (2.38)	98.17 (3.99)
**CEF**		Infusion bag	92.67 (1.35)	100.50 (2.68)	77.62 (3.67)	78.08 (3.85)	53.56 (3.16)
		Elastomeric pump	96.83 (4.03)	94.85 (3.99)	86.20 (1.53)	73.69 (3.45)	54.51 (3.63)
**CFD**		Infusion bag	102.57 (3.99)	98.62 (2.74)	83.03 (3.15)	74.69 (3.27)	61.21 (3.25)
		Elastomeric pump	103.24 (2.14)	96.48 (2.22)	70.11 (3.57)	73.00 (2.89)	59.85 (3.52)
**CAZ**		Infusion bag	100.33 (3.76)	78.30 (3.34)	70.85 (4.48)	66.94 (3.20)	58.02 (4.15)
		Elastomeric pump	98.83 (3.98)	82.06 (2.43)	77.53 (3.87)	77.06 (3.94)	54.65 (4.03)
**C/A**	Infusion bag	Ceftazidime	92.19 (1.99)	82.96 (3.85)	84.26 (2.94)	74.12 (1.69)	68.56 (2.91)
		Avibactam	100.58 (2.22)	94.18 (3.68)	94.89 (4.10)	106.09 (2.60)	94.61 (4.63)
	Elastomeric pump	Ceftazidime	94.76 (3.20)	86.60 (2.61)	77.38 (2.88)	69.83 (5.24)	67.44 (3.51)
		Avibactam	97.91 (0.52)	95.98 (4.25)	98.19 (5.72)	101.11 (2.41)	96.58 (4.69)
**C/T**	Infusion bag	Ceftolozane	101.46 (4.24)	98.08 (1.55)	106.80 (2.23)	92.15 (1.45)	78.72 (3.64)
		Tazobactam	97.16 (5.35)	98.49 (5.19)	108.28 (2.53)	105.25 (2.02)	104.11 (1.70)
	Elastomeric pump	Ceftolozane	106.19 (0.80)	97.66 (2.58)	103.92 (1.00)	95.71 (2.74)	80.53 (4.01)
		Tazobactam	102.06 (2.68)	101.01 (3.92)	95.62 (4.01)	104.61 (6.26)	98.13 (2.25)
**MRP**		Infusion bag	83.79 (3.82)	67.38 (4.16)	57.03 (2.50)	49.18 (4.75)	36.98 (3.93)
		Elastomeric pump	85.17 (2.05)	72.55 (1.85)	73.20 (3.41)	49.63 (1.98)	38.75 (3.19)
**MEV**	Infusion bag	Meropenem	79.92 (4.80)	54.25 (4.36)	51.65 (1.81)	37.95 (3.71)	17.67 (1.04)
		Vaborbactam	95.39 (2.64)	80.54 (3.77)	84.13 (0.70)	73.68 (4.02)	75.55 (4.12)
	Elastomeric pump	Meropenem	75.54 (3.46)	48.93 (3.16)	52.96 (3.08)	33.44 (4.25)	14.52 (2.13)
		Vaborbactam	101.23 (4.27)	74.55 (1.99)	76.53 (0.05)	74.75 (3.08)	72.05 (4.81)
**P/T**	Infusion bag	Piperacillin	99.73 (4.63)	105.97 (0.14)	93.33 (1.43)	93.62 (3.90)	96.02 (1.56)
		Tazobactam	103.37 (3.60)	104.05 (0.75)	94.36 (3.67)	99.64 (4.27)	97.26 (1.96)
	Elastomeric pump	Piperacillin	99.16 (5.59)	96.69 (4.89)	93.33 (1.80)	105.46 (1.26)	94.76 (4.26)
		Tazobactam	104.16 (3.45)	103.22 (1.85)	97.56 (0.09)	102.65 (0.38)	103.62 (2.11)

Abbreviations: AZT, aztreonam; CEF, cefepime; CFD, cefiderocol; CAZ, ceftazidime; C/A, ceftazidime/avibactam; C/T, ceftolozane/tazobactam; MRP, meropenem; MEV, meropenem/vaborbactam; P/T, piperacillin/tazobactam; CI, confidence interval. Unstable conditions are colored in red.

**Table 6:** Physical stability.

Antibiotic and Device		Temperature (°C)	Physical Stability			
			Color	Clearness	Precipitation	pH Range
**AZT**	Infusion bag	4	Colorless	Yes	No	5.01–4.94
		25	Colorless	Yes	No	4.91–4.88
		32	Colorless	Yes	No	5.13–5.08
		37	Colorless	Yes	No	5.00–4.96
	Elastomeric pump	4	Colorless	Yes	No	4.91–4.87
		25	Colorless	Yes	No	4.95–4.92
		32	Colorless	Yes	No	5.07–5.03
		37	Colorless	Yes	No	4.99–4.94
**CEF**	Infusion bag	4	Colorless	Yes	No	4.95–4.90
		25	From colorless to slightly yellow	Yes	No	4.99–4.67
		32	From colorless to dark yellow	Yes	No	6.83–5.08 *
		37	From colorless to slightly orange	Yes	No	7.14–4.16 *
	Elastomeric pump	4	Colorless	Yes	No	4.49–4.45
		25	From colorless to slightly yellow	Yes	No	4.95–4.90
		32	From colorless to dark yellow	Yes	No	6.66–4.55 *
		37	From colorless to slightly orange	Yes	No	7.17–4.60 *
**CFD**	Infusion bag	4	Colorless	Yes	No	5.28–5.25
		25	Colorless	Yes	No	5.48–5.11
		32	Colorless	Yes	No	6.12–5.24
		37	Colorless	Yes	No	6.51–5.31 *
	Elastomeric pump	4	Colorless	Yes	No	5.25–5.19
		25	Colorless	Yes	No	5.46–5.12
		32	Colorless	Yes	No	6.21–5.27
		37	Colorless	Yes	No	6.61–5.33 *
**CAZ**	Infusion bag	4	Colorless	Yes	No	7.10–6.65
		25	Colorless	Yes	No	7.32–6.92
		32	Colorless	Yes	No	7.27–7.06
		37	Colorless	Yes	No	7.00–6.86
	Elastomeric pump	4	Colorless	Yes	No	7.70–7.51
		25	Colorless	Yes	No	7.39–7.21
		32	Colorless	Yes	No	7.34–7.15
		37	Colorless	Yes	No	7.17–7.01
**C/A**	Infusion bag	4	Colorless	Yes	No	7.51–7.35
		25	Colorless	Yes	No	7.54–7.43
		32	Colorless	Yes	No	7.22–6.97
		37	Colorless	Yes	No	7.09–6.95
	Elastomeric pump	4	Colorless	Yes	No	7.32–7.22
		25	Colorless	Yes	No	7.47–7.07
		32	Colorless	Yes	No	7.37–7.14
		37	Colorless	Yes	No	7.21–6.98
**C/T**	Infusion bag	4	Colorless	Yes	No	5.96–5.93
		25	From colorless to slightly yellow	Yes	No	5.95–5.51
		32	From colorless to slightly yellow	Yes	No	5.88–5.62
		37	From colorless to slightly yellow	Yes	No	5.80–5.37
	Elastomeric pump	4	Colorless	Yes	No	5.97–5.86
		25	From colorless to slightly yellow	Yes	No	5.99–5.66
		32	From colorless to slightly yellow	Yes	No	5.97–5.47
		37	From colorless to slightly yellow	Yes	No	5.80–5.33
**MRP**	Infusion bag	4	Colorless	Yes	No	7.93–7.85
		25	From colorless to slightly yellow	Yes	No	7.90–7.62
		32	From colorless to slightly yellow	Yes	No	7.69–7.49
		37	From colorless to slightly yellow	Yes	No	7.64–7.34
	Elastomeric pump	4	Colorless	Yes	No	7.85–7.73
		25	From colorless to slightly yellow	Yes	No	7.92–7.73
		32	From colorless to slightly yellow	Yes	No	7.76–7.59
		37	From colorless to slightly yellow	Yes	No	7.83–7.61
**MEV**	Infusion bag	4	Colorless	Yes	No	7.92–7.85
		25	From colorless to slightly yellow	Yes	No	8.04–7.77
		32	From colorless to dark yellow	Yes	No	7.90–7.79
		37	From colorless to slightly orange	Yes	No	8.13–7.75
	Elastomeric pump	4	Colorless	Yes	No	8.18–7.99
		25	From colorless to slightly yellow	Yes	No	8.27–7.89
		32	From colorless to dark yellow	Yes	No	8.08–7.90
		37	From colorless to slightly orange	Yes	No	8.13–7.75
**P/T**	Infusion bag	4	Colorless	Yes	No	5.21–5.11
		25	Colorless	Yes	No	5.03–4.75
		32	Colorless	Yes	No	4.94–4.75
		37	Colorless	Yes	No	4.88–4.78
	Elastomeric pump	4	Colorless	Yes	No	5.46–5.36
		25	Colorless	Yes	No	5.09–4.70
		32	Colorless	Yes	No	4.96–4.71
		37	Colorless	Yes	No	4.88–4.76

Abbreviations: AZT, aztreonam; CEF, cefepime; CFD, cefiderocol; CAZ, ceftazidime; C/A, ceftazidime/avibactam; C/T, ceftolozane/tazobactam; MRP, meropenem; MEV, meropenem/vaborbactam; P/T, piperacillin/tazobactam. * indicates a change in pH of more than one unit.

**Table 7:** Stability of each antibiotic at 4, 25, 32, and 37 °C.

Temperature (°C)	AZT	CEF	CFD	CAZ	C/A	C/T	MRP	MEV	P/T
**4**	72 h	72 h	72 h	72 h	72 h	72 h	72 h	72 h	72 h
**25**	72 h	48 h	72 h	48 h	48 h	72 h	30 h	30 h	72 h
**32**	72 h	48 h	24 h	30 h	30 h	72 h	12 h	12 h	72 h
**37**	72 h	24 h	24 h	12 h	12 h	48 h	-	-	72 h

Abbreviations: AZT, aztreonam; CEF, cefepime; CFD, cefiderocol; CAZ, ceftazidime; C/A, ceftazidime/avibactam; C/T, ceftolozane/tazobactam; MRP, meropenem; MEV, meropenem/vaborbactam; P/T, piperacillin/tazobactam. Colors are in accordance with the maximum hours of stability of each antibiotic at each temperature: blue (72 h), green (48 h), pink (30 h), yellow (24 h), orange (12 h), and red (<12 h).

## Data Availability

The data presented in this study are available in this article and supplementary material.

## Supplementary Materials

The following supporting information can be downloaded at: https://www.mdpi.com/article/10.3390/pharmaceutics15122705/s1, Table S1: chromatographic conditions; Table S2: Mass spectrometry conditions.
