# Supplementary material for: Stability Studies of Antipseudomonal Beta Lactam Agents for Outpatient Therapy
Source: Pharmaceutics. 2023 Nov 30;15(12):2705. doi: 10.3390/pharmaceutics15122705 (PMC10747133; doi:10.3390/pharmaceutics15122705)
Supplement: Supplementary file 1 [file pharmaceutics-15-02705-s001.zip › pharmaceutics-2728478-supplementary.pdf]

Table S1. Chromatographic conditions

Drug	Column	Mobile phase A	Mobile phase B	Gradient	Flow rate (µL/min)	Injection volume (µL)	Total run time (min)
**Avibactam**	Kinetex F5 100 Å (2.6 µm, 150 x 2.1 mm)	2 mM AmF and 0.1% FA in MQ	0.1% FA in ACN	0-0.2 min: 4% B<br>0.2-3.5 min: gradual increase until 70% B<br>3.5-4.5 min: 70% B<br>4.50-4.51 min: descenso directo a 4% B<br>4.51-7.5 min: 4% B	300	10	7.5
**Aztreonam**	Kinetex F5 100 Å (2.6 µm, 150 x 2.1 mm)	2,5 mM AmF and 0.5% FA in MQ	0.1% FA in ACN	Isocratic elution: 85% mobile phase A and 15% mobile phase B	300	20	3
**Cefepime**	Phenomenex Luna C18 (5 µm, 150 x 2.0 mm)	2 mM AmF and 0.1% FA in MQ	0.1% FA in ACN	0-0.5 min: 0% B<br>0.5-1.5 min: gradual increase until 50% B<br>1.51-3.5 min: 95% B<br>3.51-6.0 min: 0% B	500	10	6
**Cefiderocol**	Kinetex F5 100 Å (2.6 µm, 150 x 2.1 mm)	2,5 mM AmF and 0.5% FA in MQ	0.1% FA in ACN	Isocratic elution: 74% mobile phase A and 26% mobile phase B	300	20	3
**Ceftazidime**	Kinetex F5 100 Å (2.6 µm, 150 x 2.1 mm)	5 mM AmF and 0.2% FA in MQ	0.5% FA in ACN	0-0.2 min: 4% B<br>0.2-3.5 min: gradual increase until 70% B<br>3.5-4.5 min: 70% B<br>4.50-4.51 min: descenso directo a 4% B<br>4.51-7.5 min: 4% B	300	10	7.5
**Ceftolozane**	Phenomenex Luna C18 (5 µm, 150 x 2.0 mm)	2 mM AmF and 0.1% FA in MQ	0.1% FA in ACN	0-0.5 min: 0% B<br>0.5-1.5 min: gradual increase until 50% B<br>1.51-3.5 min: 95% B<br>3.51-6.0 min: 0% B	500	10	6
**Meropenem**	Phenomenex Luna C18 (5	2 mM AmF and 0.1% FA in MQ	0.1% FA in ACN	0-0.5 min: 0% B<br>0.5-1.5 min: gradual increase until 50% B<br>1.51-3.5 min: 95% B	500	10	6

	µm, 150 x 2.0 mm)			3.51-6.0 min: 0% B			
**Piperacillin**	Phenomenex Luna C18 (5 µm, 150 x 2.0 mm)	2 mM AmF and 0.1% FA in MQ	0.1% FA in ACN	0-0.5 min: 0% B<br>0.5-1.5 min: gradual increase until 50% B<br>1.51-3.5 min: 95% B<br>3.51-6.0 min: 0% B	500	10	6
**Tazobactam**	Phenomenex Luna C18 (5 µm, 150 x 2.0 mm)	2 mM AmF and 0.1% FA in MQ	0.1% FA in ACN	0-0.5 min: 0% B<br>0.5-1.5 min: gradual increase until 50% B<br>1.51-3.5 min: 95% B<br>3.51-6.0 min: 0% B	500	10	6
**Vaborbactam**	Pursult XRs C18 (2.8 µm, 100 x 2.0 mm)	5 mM AmF and 0.2% FA in MQ	0.1% FA in ACN	0-0.5 min: 0% B<br>0.5-1.5 min: gradual increase until 50% B<br>1.51-3.5 min: 95% B<br>3.51-6.0 min: 0% B	400	10	6

Abbreviations: ACN, acetonitrile; MQ, milli-Q water; AmF, ammonium formate; FA, formic acid.

Table S2. Mass spectrometry conditions

Drug	Monitored transitions (m/z)	Ionization mode	Declustering potential (V)	Entrance potential (V)	Collision energy (V)	Cell exit potential (V)
**Avibactam**	264.001 → 95.900	Negative	-80	-10	-30	-6
**Aztreonam**	433.984 → 121.800	Negative	-80	-10	-42	-5
**Cefepime**	481.047 → 396.000	Positive	56	10	18	10
**Cefiderocol**	752.138 → 285.200	Positive	101	10	23	8
**Ceftazidime**	547.100 → 467.900	Positive	66	10	17	10
**Ceftolozane**	667.040 → 199.100	Positive	23	10	16	10
**Meropenem**	384.066 → 141.000	Positive	80	10	30	12
**Piperacillin**	518.199 → 143.200	Positive	101	10	21	12
**Tazobactam**	168.100 → 100.000	Positive	40	10	20	12
**Vaborbactam**	295.984 → 234.000	Negative	-80	-10	-30	-10
**Ampicillin (IS)**	348.123 → 207.000	Negative	-70	-10	-16	-9
**Cefixime (IS)**	454.083 → 285.000	Positive	101	10	21	8

Abbreviations: IS: internal standard.
